# Congruent and Oriented Crystallization of Mixed Sn–Pb Perovskite From the Nano‐ to Centimeter Scale

**DOI:** 10.1002/advs.202412101

**Published:** 2025-03-09

**Authors:** Donghao Miao, Zihao Zhu, Yuchen Ding, Qinyu Ning, Lei Cheng, Zheng Fang, Yi Chen, Guoping Qin, Yuedong Shi, Gang Li, Qixi Mi

**Affiliations:** ^1^ School of Physical Science and Technology and State Key Laboratory of Advanced Medical Materials and Devices ShanghaiTech University Shanghai 201210 China; ^2^ Department of Electrical and Electronic Engineering Research Institute for Smart Energy (RISE) The Hong Kong Polytechnic University Kowloon Hong Kong 999077 China

**Keywords:** crystallization kinetics, device stability, device structure, power conversion efficiency, tin‐lead perovskite solar cells

## Abstract

Metal halide perovskites have garnered widespread attention for research and applications, thanks to their high adaptability in elemental composition and optoelectronic properties. The mixed Sn–Pb perovskite FASn_0.5_Pb_0.5_I_3_ (FA^+^ is formamidinium) features a narrow bandgap of 1.25 eV, appropriate for building tandem solar cells, but faces challenges in fabricating uniform, compact films having protected surfaces and scalable dimensions. Herein, a Lewis‐base molecule trimethylthiourea (3T) is applied as a ligand to the FASn_0.5_Pb_0.5_I_3_ system, and find that it favors binding to Sn^2+^ over Pb^2+^. As a result, the tin and lead components crystallize congruently at the unit cell scale. The 3T ligand helps the FASn_0.5_Pb_0.5_I_3_ crystal grains develop into regular shapes and micron sizes, so as to fill in the film thickness and closely contact the substrate. Also, slow evaporation of 3T during annealing inhibits surface defects and renders centimeter‐wide film smoothness. Solar cells made of such FASn_0.5_Pb_0.5_I_3_ films has achieved a power conversion efficiency of 21.5% and a fill factor of 81%. Eliminating methylammonium and a hole transport layer (HTL) from these solar cells substantially boosts their short‐term and storage stabilities. These results will contribute to making streamlined, durable, and large‐area perovskite tandem solar cells.

## Graphical abstract

A Lewis‐base ligand trimethylthiourea preferentially coordinates to Sn^2+^ over Pb^2+^ and regulates the crystallization kinetics of FASn_0.5_Pb_0.5_SnI_3_. The resulting films consist of nearly stoichiometric unit cells and micrometer‐sized crystal grains exposing their (100) face. Slow annealing also contributes to film uniformity at the centimeter scale. Solar cells without a hole transport layer achieved high performance and stability.

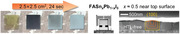

## 1 Introduction

Metal halide perovskites are versatile semiconducting materials with diverse compositions, whose exceptional optoelectronic properties have been studied extensively for solar energy, light emission, and detection applications.^[^ ^1^ ^]^ Characterized by their distinctive perovskite crystal structure, these compounds exhibit high tolerance of alloying in their constituent organic/inorganic/metal cations and halide anions, giving rise to broadly tunable bandgap energy (*E*_g_).^[^ ^2^ ^]^ Notably, mixed Sn–Pb perovskites possess narrower *E*_g_ values than their single‐metal counterparts.^[^ ^3^ ^]^ The mixed Sn–Pb perovskite FASn_0.5_Pb_0.5_I_3_ (FA^+^ is formamidinium) with *E*_g_ ≈1.25 eV, among the lowest in common perovskite materials, is well suited for building the narrow‐bandgap cell in perovskite tandem solar cells.^[^ ^4^ ^]^

To fulfill the high efficiency, low cost, and solution processibility of perovskite tandem solar cells, several issues in fabricating the narrow‐bandgap cell should be addressed. First, the FASn_0.5_Pb_0.5_I_3_ phase should be homogeneous, or else segregation of the FASnI_3_ component would result in spatial irregularities and be prone to defect formation.^[^ ^5^ ^]^ In addition, techniques incompatible with the existing wide‐bandgap cell are to be minimized, e.g., using an aqueous solution of the conducting polymer poly(3,4‐ethylenedioxythiophene): poly(styrenesulfonate) (PEDOT:PSS) as the hole transport layer (HTL).^[^ ^6^ ^]^ Moreover, extra compositions such as the volatile MA^+^ (methyl ammonium) and oxidizing NiO*_x_* may improve the initial performance, but deteriorate the reproducibility and long‐term stability of these already complex devices.^[^ ^7^ ^]^ Hence, individual cells free of MA^+^ and HTL are desirable for building tandem structures.^[^ ^6^, ^8^ ^]^

A general approach to congruent crystallization, i.e., maintaining similar compositions in the solid and solution phases, involved a ligand that balances the chemical potential of relevant metal cations, just like in the electrodeposition of metal alloys.^[^ ^9^ ^]^ In our previous work on lead‐free FASnI_3_ solar cells,^[^ ^10^ ^]^ Lewis‐base ligands were found to regulate the crystallization kinetics of FASnI_3_, thereby producing smooth and compact films, and to inhibit defect states on FASnI_3_ surfaces by chemisorption. In this article, we include trimethylthiourea (3T) in the perovskite precursor solution as an effective ligand to Sn^2+^. These advantages of Lewis‐base ligands encouraged us to investigate whether 3T would play a similar role in mixed Sn–Pb perovskite solar cells.

**Figure 1:**
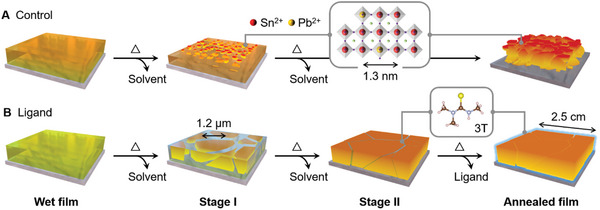
Crystallization of mixed Sn–Pb perovskite films during annealing. A) Normally, Sn^2+^‐centered octahedra (red) tend to nucleate and crystallize faster than Pb^2+^ (orange), yielding fragmented crystal grains with Sn^2+^ segregation. B) By preferentially binding to Sn^2+^, the trimethylthiourea (3T) ligand makes the perovskite layer more uniform, slowly evaporates from the annealed film, and passivates its surfaces.

Herein, we found that the 3T ligand indeed stabilized Sn^2+^ in competition with equimolar Pb^2+^. **Figure** **1** outlines several stages in the crystallization of spin‐coated FASn_0.5_Pb_0.5_I_3_ films. In the control experiment, antisolvent or heating induces rapid nucleation, whose compositions are overwhelmingly biased toward Sn^2+^ at the atomic scale. The 3T ligand, however, enables congruent crystallization of FASn_0.5_Pb_0.5_I_3_, yielding micron‐sized grains that span across the film thickness. Most of 3T can escape the annealed films as a neutral molecule, while a residual amount serves to protect the grain surfaces.^[^ ^10a^ ^]^ In this way, high‐quality FASn_0.5_Pb_0.5_I_3_ films are created over cm^2^ of area, ready to construct single‐junction or tandem solar cells.

## 2 Results and Discussion

### 2.1 Effects of Ligand on Film Formation

**Figure 2:**
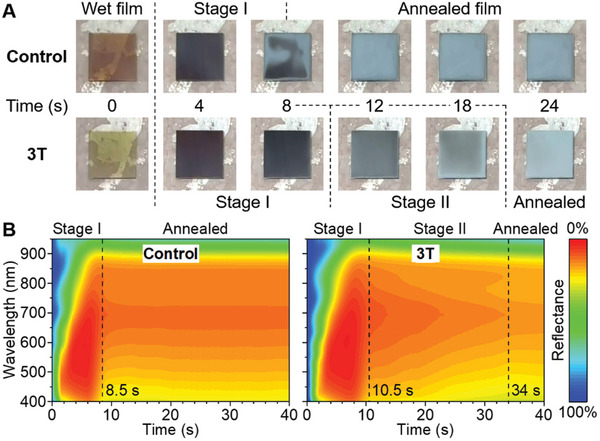
A) Still images and B) in situ reflectance spectra of FASn_0.5_Pb_0.5_I_3_ films during different stages of annealing, highlighting the regulation effect of the 3T ligand. Glass substrates are 2.5 cm in side length.

**Figure** **2** visualizes the crystallization kinetics of FASn_0.5_Pb_0.5_I_3_ films during annealing at 120 °C. Spin‐coating of a normally formulated precursor solution generated wet films that took on a brown tinge upon antisolvent rinsing, a sign of premature perovskite nucleation. When the wet film was heated, it first turned black opaque within ≈8 s (Stage I) and then quickly transitioned to matte gray. In situ reflectance spectroscopy indicated that this transition involved little change in the absorption range of the film but a rise in its reflectance to visible light. (Figure 2B) Remarkably, this transition did not take place simultaneously throughout the film's area; an irregular pattern emerged that is unrelated to spin coating or the temperature field. (Figure 2A; Video S1, Supporting Information) By contrast, including 3T in wet FASn_0.5_Pb_0.5_I_3_ films slowed them down from turning brown and black. More importantly, the presence of 3T introduced Stage II of crystallization in which the black appearance faded steadily over 15–25 s. It was possible to capture an intermediate state, e.g. at 18 s, at which the film borders displayed higher gloss (reflecting a smooth morphology) whereas the central area remained dark gray (owing to residual liquid on film surfaces). Eventually, the annealed films appeared unform, glossy gray, observed by both the camera and spectrometer.

**Figure 3:**
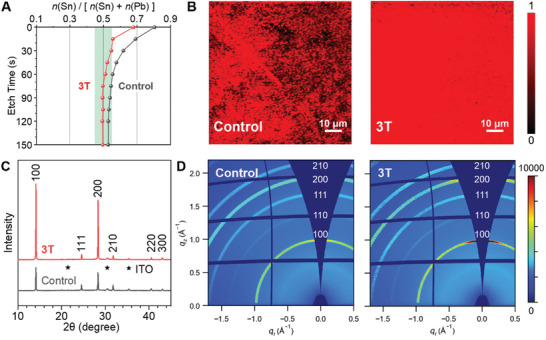
Elemental distribution and texture of FASn_0.5_Pb_0.5_I_3_ films without or with the 3T ligand. A) Depth dependence of the mole fraction of Sn^2+^. B) Distribution of crystal grains over an area of ≈0.1 × 0.1 mm^2^ in the films. C) Powder and D) Grazing Incidence XRD of the films, showing preferential (100) orientation after treatment with 3T.

The quality of annealed FASn_0.5_Pb_0.5_I_3_ films was characterized in terms of their compositional, spatial, and orientational uniformity. Elemental analysis was conducted using X‐ray Photoelectron Spectroscopy (XPS) with Ar^+^ etching at a series of surface depths. **Figure** **3**A and Table S1 (Supporting Information) show that a large excess of Sn^2+^ relative to Pb^2+^ exists at the top film surface, whereas the two elements gradually become nearly equimolar (green shaded area) at greater depths. The presence of 3T helped keep the mole fraction of Sn^2+^ ≈0.5 and thus effectively suppressed its surface segregation. Figure 3B presents Raman microscopy images of FASn_0.5_Pb_0.5_I_3_ films over an area of ≈0.1 × 0.1 mm^2^. Control samples exhibited regions of scattered signals, likely owing to uncontrolled nucleation and growth of crystal grains, but these inhomogeneous morphologies were largely smoothed out by incorporating 3T.

The effect of 3T on the texture of FASn_0.5_Pb_0.5_I_3_ films was studied by three types of X‐ray Diffraction (XRD) techniques. Powder XRD patterns in Figure 3C confirm the films to be a solid solution of a simple cubic lattice without bulk phase separation. This cubic perovskite structure was confirmed by single‐crystal XRD in a separate experiment, see the crystallographic data in Table S2 (Supporting Information). Adding 3T did not alter the crystal structure of the films, but rather intensified the 100 and 200 diffraction peaks by a factor greater than three. Figure 3D plots 2D diffraction patterns measured by Grazing Incidence (GI) XRD. By comparing the two figures, it is evident that the 100 and 200 Bragg rings were transformed from largely isotropic to preferably oriented out of plane. Furthermore, no small‐angle diffraction signals were detected at wavevector transfer *q* < 0.5 Å^−1^, which excludes 2D perovskite overlayers. Hence, both powder and GI‐XRD results agree with each other that the 3T ligand induced better crystallite alignment in FASn_0.5_Pb_0.5_I_3_ films.

**Figure 4:**
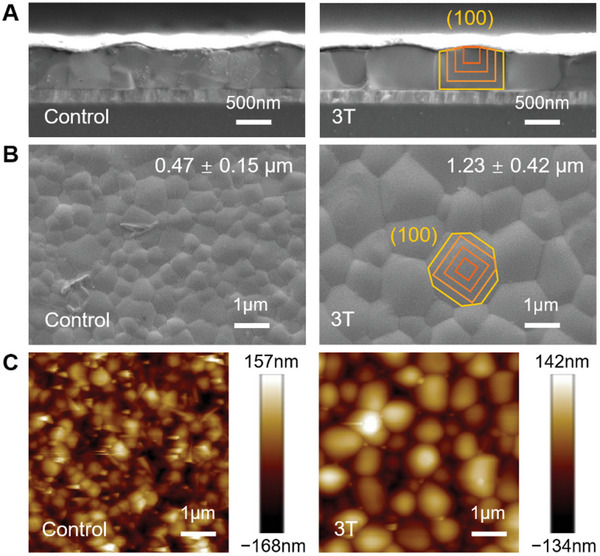
Morphology of FASn_0.5_Pb_0.5_I_3_ films without or with the 3T ligand by A,B) Scanning Electron Microscopy (SEM), and C) Atomic Force Microscopy (AFM). Contours suggest the growth course of a crystal grain. In the cross‐sectional view (A), the perovskite layer is covered by the C_60_ electron transport layer and Ag electrode.

**Figure** **4** depicts the microscopic morphology of the FASn_0.5_Pb_0.5_I_3_ films revealed by Scanning Electron Microscopy (SEM) and Atomic Force Microscopy (AFM). From the cross‐sectional view, these films had a consistent thickness of ≈0.6 µm whether or not 3T was included. FASn_0.5_Pb_0.5_I_3_ films fabricated in the control experiment consisted of crystal grains ≈0.47 µm in average size. Assuming isotropic growth rates, the film thickness could not be occupied by a single crystal grain, but comprised multiple pieces. By contrast, precursor solutions containing 3T produced grain sizes of 1.23 ± 0.42 µm, nearly three times those in the control samples. (Figure S1, Supporting Information) These columnar crystal grains not only cover the surface area completely, but also fill in the entire film thickness without fracture or stratification. (Figure 4, right column) Other imaging techniques in Figures 4C and S2 (Supporting Information) indicate the same trend that the 3T ligand helped magnify the grain sizes in FASn_0.5_Pb_0.5_I_3_ films by several folds.

### 2.2 Multiscale Mechanism for Crystal Growth

**Figure 5:**
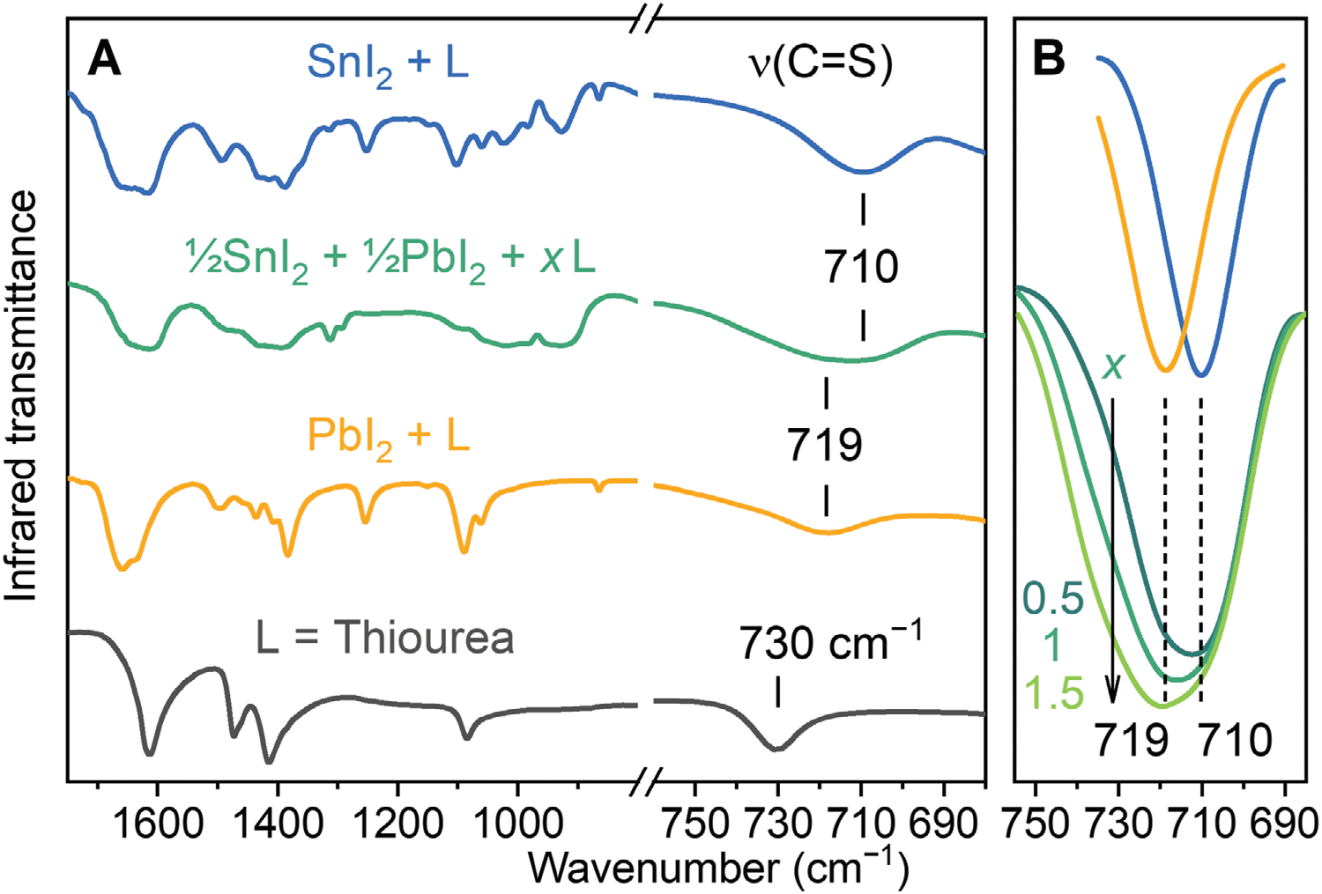
Infrared spectra of a thiourea ligand coordinated to SnI_2_, PbI_2_, or their mixture. A) Overview of the fingerprint region. B) Evolution of the C═S stretching peak as the ligand‐to‐metal ratio *x* increases, indicating favorable binding to Sn^2+^ over Pb^2+^.

To understand the chemical interactions between 3T and the metal cations, we studied υ(C═S), the characteristic stretching frequency that probes the strength of the coordination bond C═S → Sn^2+^ or Pb^2+^, in model experiments by Fourier Transform Infrared Spectroscopy (FT‐IR). **Figure** **5**A illustrates that υ(C═S) generally red‐shifts from 730 cm^−1^ for thiourea alone to 719 cm^−1^ for coordination with Pb^2+^, and then to 710 cm^−1^ for coordination with Sn^2+^. In a mixed Sn–Pb system, the overall υ(C═S) peak appears to be a superposition between υ(C═S → Sn^2+^) and υ(C═S → Pb^2+^). The relative contributions of these two components depend on the ligand‐to‐metal ratio *x*, and υ(C═S → Sn^2+^) is predominant for *x* < 1. (Figure 5B) This trend suggests that thiourea ligands prefer to associate with Sn^2+^ than Pb^2+^ under the experimental condition of *x* = ≈0.2.

The affinity of 3T toward Sn^2+^ takes effect on the crystallization process of FASn_0.5_Pb_0.5_I_3_ over three distinct length scales. In the nanometer scale of unit cell, SnI_6_ octahedra tends to self‐condensate in the mixed Sn–Pb precursor solution, forming small FASnI_3_ clusters before Pb^2+^ is mixed in.^[^ ^11^ ^]^ In the control experiment, this probably took place on the top surface of the wet FASn_0.5_Pb_0.5_I_3_ film, when it was rinsed by an antisolvent and turned brown. (Figure 2A) Premature formation of FASnI_3_ clusters become densely distributed crystal nuclei and are also responsible for surface segregation of Sn^2+^. On the other hand, 3T preferentially stabilizes individual Sn^2+^ centers and helps them blend with Pb^2+^, which promotes congruent crystallization of the film.

In the micrometer scale of the crystal grain, 3T strongly adsorbs to the growing surfaces, especially low‐index crystal planes such as {100}. This effect guides the crystal grains to grow into a cuboid shape so that their {100} faces are exposed.^[^ ^12^ ^]^ During annealing, it is conceivable that the nascent cuboid grains float on top of the wet film. The surface tension that suspends and balances these cuboid grains would also level off their (100) face. If two adjacent crystal nuclei are separated farther than the film thickness doubled, the cuboid grains would first grow to reach the film thickness and then continue expanding horizontally, until adjacent grains meet at a straight boundary. (Figure 4) Such a tiling texture minimizes void space on the bottom film surface or cracks at the grain boundary.^[^ ^13^ ^]^ Moreover, surface passivation by adsorption of 3T extends the lifetime of charge carriers in the crystal grains. (see below)

Third, 3T enhances film uniformity over the centimeter scale of device. In control experiments, the erratic pattern that emerged during annealing had a characteristic feature size of several mm. (Figure 2A; Video S1, Supporting Information) We propose that this phenomenon reflects the sensitivity of crystallization toward local solvent atmosphere and its diffusion. For example, the vapor diffusion coefficients of *N*,*N*‐dimethylformamide and dimethyl sulfoxide in air at 120 °C are approximately *D* ≈0.1 cm^2^·s^−1^,^[^ ^14^ ^]^ and their diffusion lengths in *t* = 1 s will be *L* = $\sqrt{2Dt}$ ≈0.5 cm. The 3T ligand chemisorbs to the FASn_0.5_Pb_0.5_I_3_ grain surfaces, making them no longer susceptible to the solvent vapor. Figure S3 (Supporting Information) demonstrates that substitution on three or four N–H groups of thiourea significantly weakens its intermolecular hydrogen bonding, making 3T volatile under the annealing conditions. Therefore, it is conceivable that excess 3T serves as a high‐boiling‐point (estimated 327 °C) solvent that slowly evaporates over 15–25 s in Stage II of Figure 2B, corresponding to *L* ≈2 cm, the size of the glass substrates. If the precursor solution contained a small amount (≈2 mol.%) of a non‐volatile ligand, such as unsubstituted thiourea or *N*‐methylthiourea, this ligand would survive the annealing process and protect the film surfaces.

### 2.3 Benefits of Device Performance

**Figure 6:**
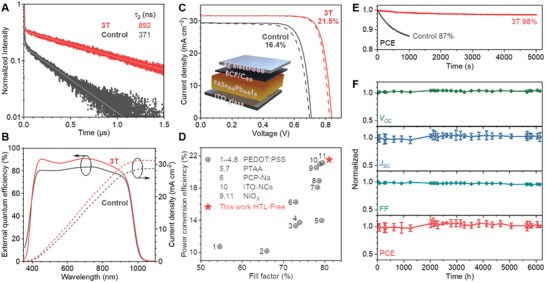
Performance enhancement of FASn_0.5_Pb_0.5_I_3_ solar cells by the 3T ligand. A) charge‐carrier lifetime. B) External quantum efficiency and the corresponding integrated current density. C) Typical current density−voltage curves and device structure (inset). D) Performance advancement of FASn_0.5_Pb_0.5_I_3_ solar cells in recent years. E) Short‐term stability in power conversion efficiency (PCE) promoted by 3T. F) Performance tracking of 3T‐treated devices during 6000 h of storage under N_2_.

**Figure** **6**A plots photoluminescence (PL) traces for the two types of films, both of which exhibited an initial fall followed by a single‐exponential decay. Adding 3T to the films extended the longer lifetime τ_2_ by 2.4 times, the same enhancement factor for their steady‐state PL intensities (Figure S4, Supporting Information), from 371 to 892 ns. The latter lifetime is among the longest reported for mixed Sn–Pb perovskites.^[^ ^4a^ ^]^ Complete solar cell devices were fabricated using the inverted planar structure ITO/FASn_0.5_Pb_0.5_I_3_/C_60_/Ag, where the perovskite layer directly contacted the indium tin oxide (ITO) conducting substrate without a hole‐transport layer (HTL) in between. Figures 6C and S5A (Supporting Information) show the current density–voltage (*J*–*V*) characteristics of excellent devices under simulated AM1.5G sunlight, and the performance parameters are summarized in Table S3 and Figure S5 (Supporting Information). Treating the perovskite layer with 3T and a small amount of thiourea raised the open‐circuit voltage (*V*_OC_) of the devices significantly from 0.71 to 0.84 V, which also lifted the fill factor (FF) from 78% to 81%. The short‐circuit current density (*J*_SC_) increased from 28.9 to 31.6 mA·cm^−2^, agreeing with the elevated light absorption (Figure S6, Supporting Information) and external quantum efficiency (EQE, Figure 6B) curves. PCE of the champion device grew from 16.4% to 21.5%. We further fabricated devices with centimeter‐sized electrodes. (Figure S7A, Supporting Information) Illumination over 1 cm^2^ generated *V*_oc_ and *J*_sc_ similar to those of small‐area devices, whereas FF dropped below 79% likely owing to increased series resistance, realizing a PCE of 20.9%. Photocurrent mapping (Figure S7B, Supporting Information) suggests that the photoresponse varies by only ≈1% over the active area of 0.9 × 1.4 cm^2^. Figure 6D and Table S3 (Supporting Information) survey the recent progress in FASn_0.5_Pb_0.5_I_3_ solar cells.^[^ ^4^, ^7^ ^]^ By adopting a more concise, HTL‐free device structure, we nonetheless achieved leading performance in terms of FF and PCE. Including an HTL by using PEDOT slightly increased *V*_oc_ but also lowered *Jsc* and FF, ineffective to the overall performance.

Stability tests were carried out in both short and long terms. In Figure 6E, a FASn_0.5_Pb_0.5_I_3_ solar cell containing 3T operated steadily under 5000 s of continuous simulated sunlight. A relative decay of 2% in power output during the first 2000 s likely resulted from a temperature rise of the black test fixture. In the control experiments, however, the normalized PCE dropped by ≈1/8 within only 1000 s and would decline further. Figure 6F tracks the performance data of unencapsulated devices over 6000 h of storage under N_2_, and *V*_OC_, *J*_SC_, FF, and PCE were tested intermittently during this period. After 2000 h, the solar simulator was installed a new Xe light bulb and *J*_SC_ became slightly higher. Nonetheless, fluctuations of these key performance parameters stayed within ≈3% around a long‐standing mean value. Treatment by 3T also rendered the perovskite films much higher resistance toward air and moisture. (Figure S8, Supporting Information)

The above results corroborate the multiple benefits of the 3T ligand. First, it helps afford chunky FASn_0.5_Pb_0.5_I_3_ crystal grains that densely pack the perovskite layer, which promote light absorption as well as charge‐carrier collection and thus improve *J*_sc_ and FF. Second, these columnar crystal grains create a large contact area with the ITO substrate, making an HTL unnecessary. The absence of an HTL also avoids complications such as parasitic light absorption and interfacial degradation. Last but not least, 3T protects the grain surfaces from electronic or chemical defects. Consequently, the *V*_oc_ and stability of the solar cells are substantially enhanced.

## 3 Conclusion

In summary, we investigated the influence of trimethylthiourea (3T) on the crystallization process and device performance of FASn_0.5_Pb_0.5_I_3_ solar cells. Evolution of the υ(C═S) peak in the infrared spectra suggests that 3T as an additive favorably binds to Sn^2+^ over Pb^2+^, which suppressed early nucleation of a Sn‐rich phase when the wet film was annealed. Instead, nearly stoichiometric FASn_0.5_Pb_0.5_I_3_ crystallized at a slower rate, allowing the crystal grains to develop their natural (100) face and become preferentially oriented. Growth of the crystal grains proceeds first downward to reach the substrate, and then laterally to close the gap between adjacent grains. Excess 3T evaporated gradually in Stage II of the annealing process, leaving a centimeter‐wide smooth film with passivated surfaces. As a result, photovoltaic devices of the inverted, HTL‐free structure gained a PCE of 21.5% over 0.04 cm^2^ illumination area and 20.9% over 1 cm^2^, and are particularly well performing in terms of charge‐carrier lifetime, fill factor, and stability. Next, we will fine‐tune the thickness of the FASn_0.5_Pb_0.5_I_3_ layer and its top and bottom interfaces, in order to optimize *V*_oc_ and *J*_sc_, before integrating with a wide‐bandgap device to create a tandem solar cell. The crystallization mechanism studied herein should also be valid for larger‐area films from other coating methods.

## Conflict of Interest

The authors declare no conflict of interest.

## Author Contributions

D.M. and Z.Z. contributed equally to this work. Z.Z. and D.M. conceived and executed the experiments. D.M., Z.Z., and L.C. fabricated the perovskite films and devices, and D.M. performed structural, morphological, and photovoltaic characterizations. Y.D. synthesized raw materials and conducted in situ optical spectroscopies and quantum efficiency measurements. Q.N. contributed to Atomic Force Microscopy and video recording. Z.F. grew the single crystal and Y.C. collected and resolved its structural data. G.Q. contributed to X‐ray Photoelectron Spectroscopy. Y.S. measured steady‐state absorption and photoluminescence spectra. Q.M. and G.L. acquired and built lab‐specific instruments and supervised the research project. Q.M. and D.M. analyzed and visualized the experimental results. Q.M., D.M., and Z.Z. wrote and revised this article.

## Supporting information



Supporting Information

Supplemental Video 1

## Data Availability

The data that support the findings of this study are openly available in CCDC at https://www.ccdc.cam.ac.uk/data_request/cif, reference number 2376240.
