# Supplementary material for: Congruent and Oriented Crystallization of Mixed Sn–Pb Perovskite From the Nano‐ to Centimeter Scale
Source: Adv Sci (Weinh). 2025 Mar 9;12(17):2412101. doi: 10.1002/advs.202412101 (PMC12061251; doi:10.1002/advs.202412101)
Supplement: Supplementary file 1 — Supporting Information [file ADVS-12-2412101-s001.docx]

Supporting Information

Congruent and Oriented Crystallization of Mixed Sn–Pb Perovskite from the Nano- to Centimeter Scale

Donghao Miao^1^,† Zihao Zhu^1^,† Yuchen Ding^1^, Qinyu Ning^1^, Lei Cheng^2^, Zheng Fang^1^, Yi Chen^1^, Guoping Qin^1^, Yuedong Shi^1^, Gang Li^2^, and Qixi Mi^1^*

**Experimental section**

**Materials**

All solvents and compounds were stored and handled in N_2_ gloveboxes (Vigor Tech) containing <0.1 ppm O_2_ and H_2_O at 25–28 °C.

*N,N*-dimethylformamide (DMF, 99.8% SuperDry, J&K Scientific 983353), dimethyl sulfoxide (DMSO, 99.9% SuperDry, J&K Scientific 935690), isopropanol (IPA, 99% SuperDry, J&K Scientific 919270), formamidinium iodide (FAI, Tokyo Chemical Industry F0974), Tin(II) iodide (SnI_2_, 5N, Shanghai Huiyi T209), lead(II) iodide (PbI_2_, 99.99%, Tokyo Chemical Industry L0279), thiourea (99%, Macklin T819602), ethylenediamine dihydroiodide (EDAI_2_, 98%, Tokyo Chemical Industry E1222), fullerene C_60_ (99.9%, Xi’an Yuri Solar 305008), and bathocuproine (BCP, >98%, Adamas 58984B) were obtained commercially. Toluene (>99.5%, Sinopharm 10022818) was distilled in the presence of Na–K alloy and benzophenone ketyl inside the N_2_ glovebox. Trimethyl­thiourea (3T, 98%, Macklin T849530) was sublimed at 75 °C, ~1 Pa before use.

**Precursor solutions**

Precursor solutions containing nominally 1.8 M FASn_0.5_Pb_0.5_I_3_ were prepared by dissolving FAI (309.6 mg, 1.8 mmol), SnI_2_ (335 mg, 0.900 mmol), PbI_2_ (415 mg, 0.900 mmol), 3T (44 mg, 0.44 mmol), and thiourea (3 mg, 0.04 mmol) in 0.75 mL DMF and 0.25 mL DMSO, and stirring overnight. In control experiments, 3T and thiourea were omitted. The resulting clear, bright yellow solution was filtered through a 0.22-μm PTFE filter before use.

**Fabrication and Evaluation of Solar Cells**

Indium tin oxide (ITO) conducting glass slides (Advanced Election Technology Co., Ltd., 8 Ω·🞏^−1^, 2.5 × 2.5 cm^2^) were cleaned and treated with ultraviolet light and ozone (SETCAS SC-UV-I) for 20 min. Inside a N_2_ glovebox (Vigor Tech), the perovskite precursor solution (90 μL) was spin-coated (Sawatec SM-150) at 1000 rpm for 10 s and then 5000 rpm for 40 s. Crystallization of the perovskite layer was promoted by a toluene rinse (500 μL) during the second step of spin coating, and then annealing at 120 °C for 10 min. A saturated solution (60 μL) of ethylenediamine dihydroiodide in isopropanol was spin-coated on top of the perovskite layer at 6000 rpm for 30 s, followed by annealing at 100 °C for 1 min. Layers of C_60_ (25 nm), bathocuproine (5 nm), and patterned Ag electrodes (80 nm) were successively formed under high vacuum in a thermal evaporator (Anhui Jiashuo JSD400) attached to the N_2_ glovebox.

***Current density–voltage (J–V)*** *characteristics* of the solar cells were measured between −0.1 and 0.9 V at steps of 0.01 V using a Keithley 2450 source/meter, under 100 mW·cm^-2^ of simulated AM1.5G illumination (Zolix Sirius-SS150A) through a 0.20 × 0.20 cm^2^ (regular) or 0.80 × 1.25 cm^2^ (large area) mask.

***External quantum efficiencies (EQE)*** were measured using a tunable mono­chromatic light source (Zolix TLS3-T150A), projected onto solar cells in the glovebox via a feed-through optical fiber. Under zero bias voltage, the wavelength-dependent photocurrents were monitored by a Keithley 2450 source/meter, and referenced to a calibrated Si solar cell (Zolix QE-B3) with matching spectral range. Integrated photocurrent density was derived from experimental EQEs and the standard AM1.5G spectrum.

***Photocurrent mapping*** was performed by placing the solar cell on an XY translation stage (Zolix SC300-2A) under a 5X objective lens. As the solar cell moved relative to the focused 532 nm laser point, photocurrent registered on a Keithley 2450 source/meter under zero bias voltage and formed a raster image on a computer.

**X-ray Photoelectron Spectroscopy (XPS)**

Perovskite films on ITO glass slides were transferred from the N_2_ glovebox by a vacuum transfer module to a photoelectron spectrometer (Thermo Scientific ESCALAB 250Xi). After various times of Ar^+^ etching, high-resolution spectra for Sn 3*d* and Pb 4*f* were taken and analyzed by the Thermo Avantage software. (Table S1)

**X-ray Diffraction (XRD)**

***Powder XRD*** patterns of spin-coated FASn_0.5_Pb_0.5_I_3_ films on ITO glass slides were acquired on a Bruker D2 Phaser emitting Cu *K*α radiation (λ = 1.5418 Å) under ambient conditions.

***Grazing-Incidence XRD*** data were recorded at the BL16B1 beamline of Shanghai Synchrotron Radiation Facility (SSRF). The photon energy was 10 keV at an incident angle of 1.0° and the diffracted X-rays were collected by a Pilatus area detector. Raw diffraction data was processed and plotted by the GIWAXS Tools software.

***Single-crystal XRD*** was performed on a Bruker D8 VENTURE diffracto­meter with Mo *K*α radiation (λ = 0.71073 Å) at 282K. The diffraction data were handled by the Olex2 software, and the crystal structures were solved and refined by the ShelXT and ShelXL programs (Table S2).

**Microscopy**

Top-view and cross-sectional micrographs of annealed FASn_0.5_Pb_0.5_I_3_ films on ITO substrates were imaged by a Field-Emission *Scanning Electron Microscope* (JEOL JSM-7800F), under an accelerating voltage of 1 kV and a working distance of 8 mm. Grain sizes were outlined and quantified by the ImageJ software.

***Atomic Force Microscopy (AFM)*** was carried out on a high-speed model (Bruker Dimension FastScan) in the tapping mode under ambient conditions, using RTESPA-300-30 probes (MikroMasch). Images were collected in the range of 10 × 10 μm^2^ (512 × 512 pixels) and processed by the AutoMET software.

**Spectroscopic Mapping**

***Raman Mapping*** was performed on a Scanning Nearfield Optical Microscope (WITec GmbH alpha 300 RS), under the conditions of 532 nm excitation and 70–150 cm^-1^ shift wavenumbers. Images were collected in the range of 85 × 85 μm^2^ (170 × 170 pixels) and processed by the Project FIVE software.

***Photoluminescence Mapping*** utilized a Confocal Laser Scanning Microscope (Olympus FV3000). Laser excitation (561 nm) was directed from below the glass substrate and scanned in the XY focal plane. PL signals were received by two photomultiplier tubes after the detection pinhole, and collected in the range of 125 × 125 μm^2^ (4096 × 4096 pixels). (Figure S2)

**Photoluminescence (PL)**

*Steady-state PL* spectra were collected on a Horiba Fluorolog-3 fluorimeter using 500 nm excitation and a CCD detector under ambient conditions. (Figure S4) *PL lifetimes* were obtained by using the time-correlated single-photon counting (TCSPC) method on a Horiba DeltaFlex picosecond fluorimeter excited by a pulsed DeltaDiode at 675 nm. Near-infrared emission was detected by a cooled photomultiplier tube (Hamamatsu R5509-73) with an instrumental response function (IRF) about 5 ns wide.

**Steady-State Spectroscopy**

In the glovebox, SnI_2_, PbI_2_, or both (1.0 mmol each or in total) and thiourea (0.5–1.5 mmol) were dissolved in DMF (1.0 mL). The filtered solution (50 μL) was spread on disposable KBr discs (Ø13 mm) and dried under vacuum overnight. *Fourier Transform Infrared Spectra (FT-IR)* of the coordination complexes were acquired on a Bruker Vertex 70 spectrometer. Spectral baselines were removed for convenience in comparing different sets of data.

*Absorption Spectra* of annealed FASn_0.5_Pb_0.5_I_3_ films were recorded in the transmission mode by an Agilent Cary 5000 spectrophotometer. Absorption background due to the ITO substrate was subtracted from the raw data. (Figure S5)

***In Situ* Reflectance Spectroscopy**

In the N_2_ glovebox, a white ceramic plate was heated to 120 °C and illuminated by two incandescent light bulbs (25 W, Osram Spot R63). Before and after a wet sample film was placed, the reflected light entering an overhead optical fiber (SUH400, NA 0.22) was recorded by a spectrometer (IdeaOptics PG2000 Pro) at 500 ms integration periods. The time-dependent spectra were calculated according to

$$\text{R}\text{ }=\text{ }\frac{\text{S}\text{ }-\text{ }\text{D}}{\text{L}\text{ }-\text{ }\text{D}}\times\text{100\%}$$

where *R*, *S*, *D*, and *L* stand for reflectance, sample, dark and lamp spectra, respectively.

**Data and code availability**

The Crystallographic Information Files (CIFs) for structures reported in this study have been deposited at the Cambridge Crystallographic Data Centre (CCDC), under deposition number 2376240. These data can be obtained free of charge from The Cambridge Crystallographic Data Centre via www.ccdc.cam.ac.uk/data_request/cif.

**Figure S1**. Distribution of SEM grain sizes in FASn_0.5_Pb_0.5_I_3_ films with or without 3T. (Figure 4B)

**
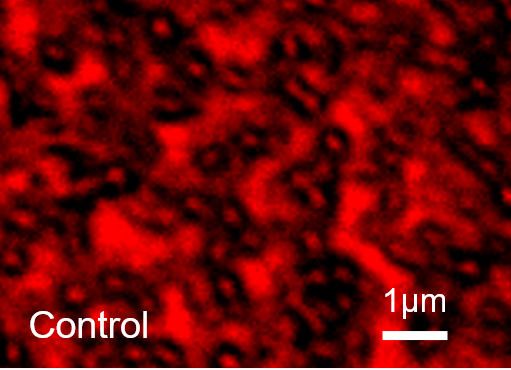
**

**
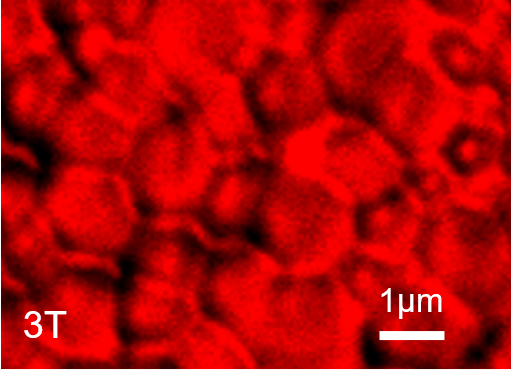
**

**Figure S2.** Photoluminescence mapping of FASn_0.5_Pb_0.5_I_3_ films with or without 3T.


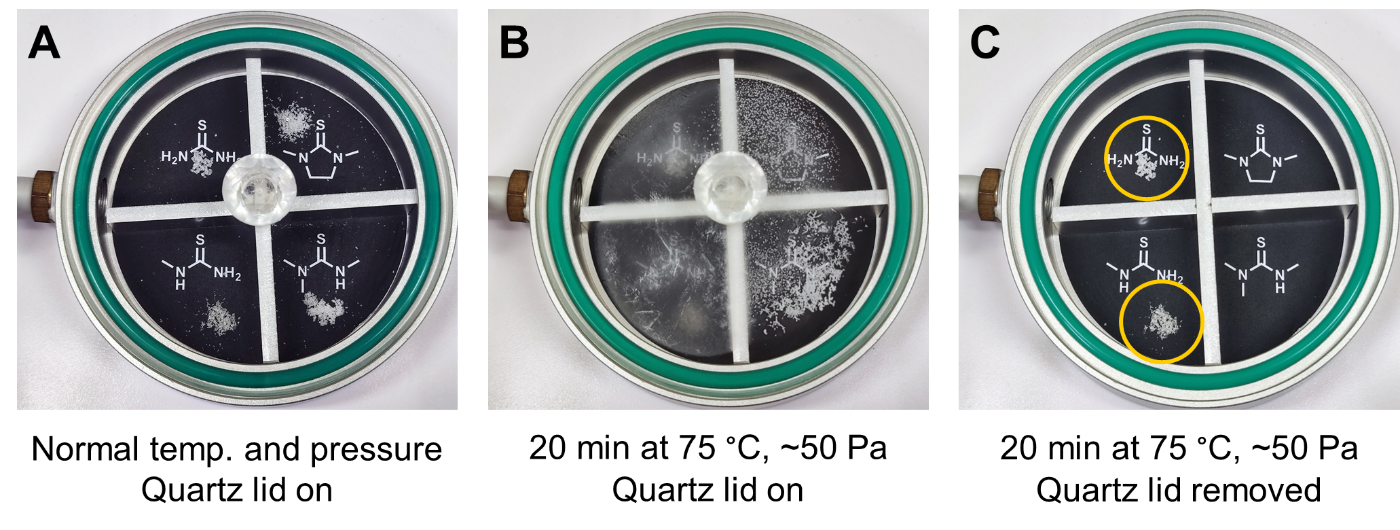

**Figure S3.** Distinct volatilities for substituted thiourea ligands. (A–C) Thiourea (T), *N*-methylthiourea (1T), trimethylthiourea (3T), and *N*,*N*′-dimethylethylenethiourea (DMETU) in counterclockwise order were placed in a vacuum chamber (Ø10 cm) with a quartz lid. After evacuation at 75 °C and ~50 Pa, 3T and DMETU sublimed completely whereas T and 1T persisted (yellow circles). (D) Boiling point of substituted urea and thiourea compounds. *^a^* Sci­Finder. *^b^* Reaxys. *^c^* Converted from reduced pressure to atmospheric. *^d^* Estimate in this work.

**Figure S4.** Photoluminescence spectra of FASn_0.5_Pb_0.5_I_3_ films with or without 3T.

**Figure S5.** Performance enhancement for FASn_0.5_Pb_0.5_I_3_ solar cells by adding the thiourea (T) and trimethyl­thiourea (3T) ligands individually or both. (A) *J*–*V* characteristics of champion devices under various fabrication conditions. (B–E) Statistics of open-circuit voltage (*V*_oc_), short-circuit current (*J*_sc_), fill factor (FF), and power conversion efficiency (PCE).

**Figure S6.** Absorption spectra of FASn_0.5_Pb_0.5_I_3_ films with or without 3T near the band­gap energy.


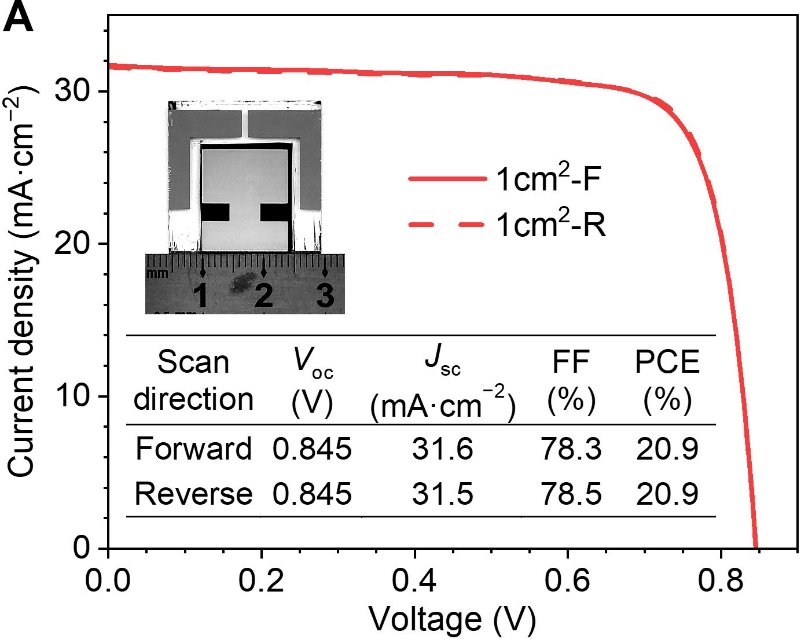

**Figure S7.** A large-area FASn_0.5_Pb_0.5_I_3_ solar cell fabricated with 3T. (A) Photograph of the device (inset) and its performance under 1 cm^2^ illumination. (B) Photocurrent mapping over the active area of 0.9 × 1.4 cm^2^ reveals good spatial uniformity. The step size in both *x* and *y* directions was 0.02 nm.


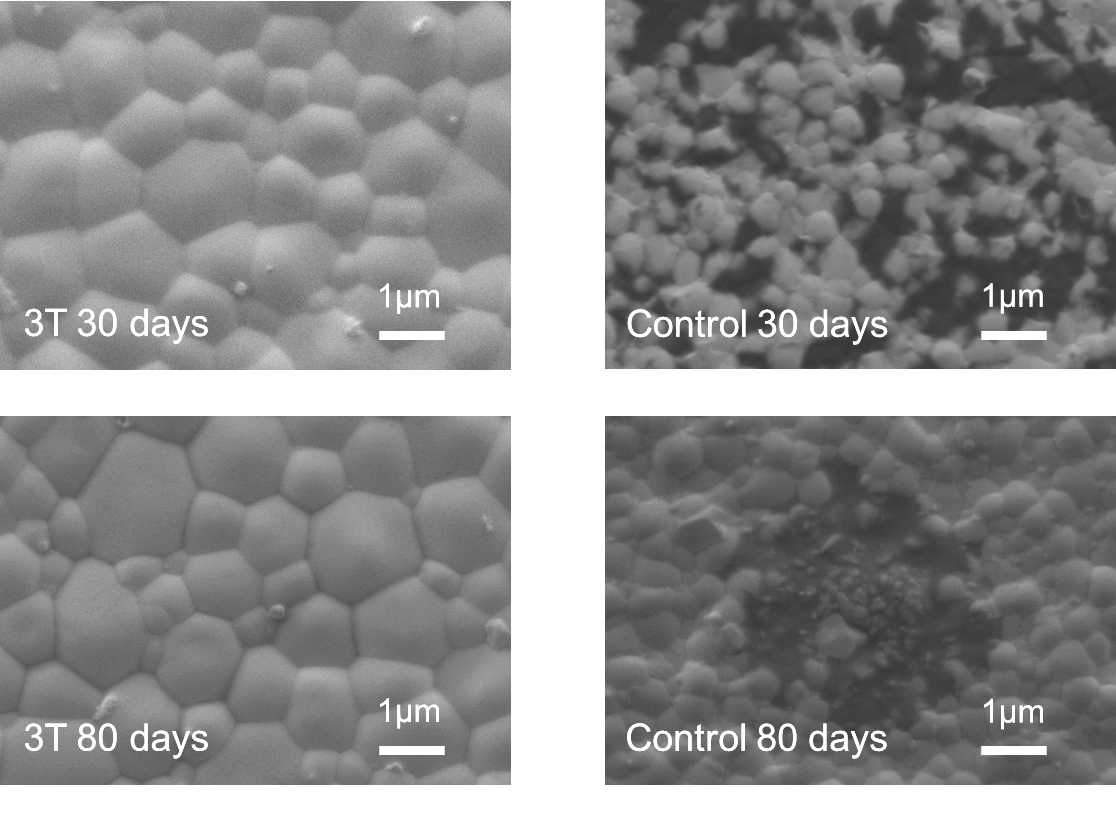


**Figure S8.** SEM images of FASn_0.5_Pb_0.5_I_3_ films with or without 3T, after exposure to air for 30 and 80 days.

**Table S1.** Mole fraction *x*(Sn) in FASn_0.5_Pb_0.5_I_3_ films with or without 3T, reflected by X-ray Photoelectron Spectra (XPS) at various etching depths.

| Etch Time (s) | **Control** | | | **3T** | | |
| --- | --- | --- | --- | --- | --- | --- |
|  | Sn 3*d* area (10^5^) | Pb 4*f* area (10^5^) | *x*(Sn) | Sn 3*d* area (10^5^) | Pb 4*f* area (10^5^) | *x*(Sn) |
| 0 | 4.4 | 1.1 | 0.80 | 2.5 | 1.2 | 0.68 |
| 15 | 4.1 | 1.8 | 0.69 | 2.7 | 2.2 | 0.55 |
| 30 | 3.5 | 2.2 | 0.61 | 2.8 | 2.3 | 0.55 |
| 45 | 3.2 | 2.3 | 0.58 | 2.7 | 2.4 | 0.53 |
| 60 | 3.0 | 2.4 | 0.56 | 2.6 | 2.5 | 0.51 |
| 75 | 3.0 | 2.5 | 0.55 | 2.5 | 2.5 | 0.50 |
| 90 | 2.9 | 2.5 | 0.54 | 2.5 | 2.5 | 0.50 |
| 105 | 2.9 | 2.5 | 0.54 | 2.5 | 2.5 | 0.50 |
| 120 | 2.9 | 2.5 | 0.54 | 2.5 | 2.5 | 0.50 |
| 150 | 2.8 | 2.5 | 0.53 | 2.5 | 2.5 | 0.50 |
| 180 | 2.9 | 2.6 | 0.53 | 2.5 | 2.5 | 0.50 |

**Table S2**. Crystallographic data and structural refinement for a FASn_0.5_Pb_0.5_I_3_ single crystal.

| Formula | CN_2_Sn_0.5_Pb_0.5_I_3_ (FW = 583.67) |
| --- | --- |
| Temperature (K) | 282 |
| Crystal system | Cubic |
| Space group | *Pm*−3*m* |
| *a, b, c* (Å) | 6.3527(3) |
| α, β, γ (°) | 90 |
| *V* (Å^3^) | 256.37(4) |
| *Z* | 1 |
| ρ_calc_ (g·cm^-3^) | 3.780 |
| μ (mm^-1^) | 18.428 |
| *F*(000) | 245.0 |
| Radiation | Mo *K*α (λ = 0.71073) |
| 2θ range for data collection (°) | 6.414 to 54.06 |
| Index ranges | −8 ≤ *h* ≤ 8, −8 ≤ *k* ≤ 8, −7 ≤ *l* ≤ 8 |
| Reflections collected | 3303 |
| Independent reflections | 85 [*R*_int_ = 0.0808, *R*_sigma_ = 0.0227] |
| Data/restraints/parameters | 85 / 0 / 9 |
| Goodness-of-fit on *F*^2^ | 1.259 |
| Final *R* indexes [*I* ≥ 2σ(*I*)] | *R*_1_ = 0.0401, *wR*_2_ = 0.1109 |
| Final *R* indexes [all data] | *R*_1_ = 0.0412, *wR*_2_ = 0.1120 |
| Largest difference peak/hole (e·Å^−3^) | 1.47 / −1.62 |

**Table S3.** Performance parameters of champion FASn_0.5_Pb_0.5_I_3_ solar cells in this work.

| Device | Scan direction | *V*_oc_  (V) | *J*_sc_  (mA·cm^−2^) | FF (%) | PCE (%) | Hysteresis (%) |
| --- | --- | --- | --- | --- | --- | --- |
| Control | Forward | 0.71 | 29.4 | 77.5 | 16.2 | 5.1 |
|  | Reverse | 0.70 | 29.4 | 74.6 | 15.4 |  |
| 3T | Forward | 0.84 | 31.6 | 81.1 | 21.5 | 1.4 |
|  | Reverse | 0.83 | 31.6 | 80.7 | 21.2 |  |

**Table S4.** Performance of FASn_0.5_Pb_0.5_I_3_ solar cells reported in recent years.

| Year | PCE (%) | *V*_oc_ (V) | *J*_sc_  (mA·cm^−2^) | FF (%) | Hole Trans-port Layer | Ref. |
| --- | --- | --- | --- | --- | --- | --- |
| 2016 | 10.2 | 0.70 | 21.9 | 66 | PEDOT:PSS | ^[1]^ |
| 2017 | 10.76 | 0.695 | 28.37 | 54.6 | PEDOT:PSS | ^[2]^ |
| 2018 | 16.27 | 0.73 | 28.51 | 73 | PCP-Na | ^[3]^ |
| 2019 | 13.98 | 0.72 | 24.5 | 79.3 | PTAA | ^[4]^ |
| 2020 | 13.33 | 0.69 | 26.56 | 73 | PEDOT:PSS | ^[5]^ |
| 2020 | 18.1 | 0.76 | 30.3 | 78.3 | PTAA | ^[6]^ |
| 2022 | 13.74 | 0.69 | 26.93 | 74 | PEDOT:PSS | ^[7]^ |
| 2022 | 18.9 | 0.805 | 29.8 | 78.7 | PEDOT:PSS | ^[8]^ |
| 2022 | 21.0 | 0.841 | 31.7 | 78.9 | ITO NCs | ^[8]^ |
| 2023 | 20.53 | 0.846 | 31.09 | 78 | NiO*_X_* | ^[9]^ |
| 2024 | 21.12 | 0.846 | 31.39 | 79.5 | NiO*_X_* | ^[10]^ |
| 2024 | 21.5 | 0.84 | 31.6 | 81.1 | None | This work |

**References**

[1] G. E. Eperon, T. Leijtens, K. A. Bush, R. Prasanna, T. Green, J. T.-W. Wang, D. P. McMeekin, G. Volonakis, R. L. Milot, R. May, A. Palmstrom, D. J. Slotcavage, R. A. Belisle, J. B. Patel, E. S. Parrott, R. J. Sutton, W. Ma, F. Moghadam, B. Conings, A. Babayigit, H.-G. Boyen, S. Bent, F. Giustino, L. M. Herz, M. B. Johnston, M. D. McGehee, H. J. Snaith, *Science* **2016**, 354, 861.

[2] J. Liu, G. Wang, Z. Song, X. He, K. Luo, Q. Ye, C. Liao, J. Mei, *J. Mater. Chem. A* **2017**, 5, 9097.

[3] S. Shao, Y. Cui, H. Duim, X. Qiu, J. Dong, G. H. ten Brink, G. Portale, R. C. Chiechi, S. Zhang, J. Hou, M. A. Loi, *Adv. Mater.* **2018**, 30, 1803703.

[4] A. M. Igual-Muñoz, J. Ávila, P. P. Boix, H. J. Bolink, *Sol. RRL* **2019**, 4, 1900283.

[5] M. T. Klug, R. L. Milot, J. B. Patel, T. Green, H. C. Sansom, M. D. Farrar, A. J. Ramadan, S. Martani, Z. Wang, B. Wenger, J. M. Ball, L. Langshaw, A. Petrozza, M. B. Johnston, L. M. Herz, H. J. Snaith, *Energy Environ. Sci.* **2020**, 13, 1776.

[6] C. Park, J. Choi, J. Min, K. Cho, *ACS Energy Lett.* **2020**, 5, 3285.

[7] L. Wang, Z. Wang, H. Li, B. Chang, L. Pan, Z. Xie, L. Yin, *ACS Appl. Mater. Interfaces* **2022**, 14, 18302.

[8] P. Wu, J. Wen, Y. Wang, Z. Liu, R. Lin, H. Li, H. Luo, H. Tan, *Adv. Energy Mater.* **2022**, 12, 2202948.

[9] Y. Zhou, Z. Wang, J. Jin, X. Zhang, J. Zou, F. Yao, Z. Zhu, X. Cui, D. Zhang, Y. Yu, C. Chen, D. Zhao, Q. Cao, Q. Lin, Q. Tai, *Angew. Chem. Int. Ed.* **2023**, 62, 2300759.

[10] Y. Zhou, T. Guo, J. Jin, Z. Zhu, Y. Li, S. Wang, S. Zhou, Q. Lin, J. Li, W. Ke, G. Fang, X. Zhang, Q. Tai, *Energy Environ. Sci.* **2024**, 17, 2845.
